# Virtual Non-Contrast versus True Non-Contrast Computed Tomography: Initial Experiences with a Photon Counting Scanner Approved for Clinical Use

**DOI:** 10.3390/diagnostics11122377

**Published:** 2021-12-16

**Authors:** Julius Henning Niehoff, Matthias Michael Woeltjen, Kai Roman Laukamp, Jan Borggrefe, Jan Robert Kroeger

**Affiliations:** 1 Department of Radiology, Neuroradiology and Nuclear Medicine, Johannes Wesling University Hospital, Ruhr University Bochum, 44801 Bochum, Germany; MatthiasMichael.Woeltjen@muehlenkreiskliniken.de (M.M.W.); Jan.Borggrefe@muehlenkreiskliniken.de (J.B.); JanRobert.Kroeger@muehlenkreiskliniken.de (J.R.K.); 2 Department of Diagnostic and Interventional Radiology, Faculty of Medicine, University Hospital Cologne, University of Cologne, 50923 Cologne, Germany; kai.laukamp@uk-koeln.de

**Keywords:** computed tomography, photon counting detector, virtual non-contrast, material decomposition, iodine quantification

## Abstract

The present study evaluates the diagnostic reliability of virtual non-contrast (VNC) images acquired with the first photon counting CT scanner that is approved for clinical use by comparing quantitative image properties of VNC and true non-contrast (TNC) images. Seventy-two patients were retrospectively enrolled in this study. VNC images reconstructed from the arterial (VNCa) and the portalvenous (VNCv) phase were compared to TNC images. In addition, consistency between VNCa and VNCv images was evaluated. Regions of interest (ROI) were drawn in the following areas: liver, spleen, kidney, aorta, muscle, fat and bone. Comparison of VNCa and VNCv images revealed a mean offset of less than 4 HU in all tissues. The greatest difference between TNC and VNC images was found in spongious bone (VNCv 86.13 HU ± 28.44, *p* < 0.001). Excluding measurements in spongious bone, differences between TNC and VNCv of 10 HU or less were found in 40% (VNCa 36%) and differences of 15 HU or less were found in 72% (VNCa 68%) of all measurements. The underlying algorithm for the subtraction of iodine works in principle but requires adjustments. Until then, special caution should be exercised when using VNC images in routine clinical practice.

## 1. Introduction

Non-enhanced computed tomography (NCCT) images generally provide limited soft tissue contrast. Thus, contrast-enhanced computed tomography (CECT) scans offer a higher diagnostic value for most clinical questions. Consequently, the number of NCCT scans has decreased after the introduction of intravenous contrast agents—especially in abdominal imaging. Nevertheless, certain findings still require a NCCT scan for a reliable evaluation. Typical examples include the assessment of adrenal gland lesions or the evaluation of hepatic steatosis [1,2].

In recent years, the introduction of the dual energy CT (DECT) and the evolving post-processing capabilities have been a great step forward in CT technology [3]. DECT scanners make use of two different energy spectra in order to obtain separate attenuation profiles for lower and higher energy photons. Technically, the DECT concept can be implemented in different ways: Rapid kVp-switching CT (tube voltage changes rapidly between low and high kVp), dual source CT (two tube-detector pairs), split beam CT (tube output is filtered and detector rows are read out separately) or spectral detector CT (two scintillator layers). Tube voltages typically range from 80 kVp up to 140 kVp. By obtaining separate attenuation profiles with DECT scanners, it is possible to decompose materials, e.g., iodine. This, in turn, offers the possibility to subtract the iodine-associated attenuation from CECT images resulting in virtual non-contrast images (VNC) [4,5,6]. Numerous clinical studies have proven that VNC images obtained with spectral CTs are diagnostically reliable [7,8,9,10,11,12,13]. It seems conceivable that VNC images could potentially replace true non-enhanced phases in the future [7,14,15].

In 2021, CT technology has taken another major step forward with the introduction of the first clinical CT scanner that makes use of a photon counting detector (PCD) with quantum technology to increase spectral imaging capabilities (Photon Counting CT, PCCT, Naeotom Alpha, Siemens Healthineers, Erlangen, Germany).

PCDs consist of a single thick layer of a semiconductor material and can convert x-rays directly into an electrical signal—unlike conventional CT detectors that require an additional step by converting x-rays into visible light first. Furthermore, PCDs are able to detect individual photons and their associated energy. Conventional CT detectors, also known as energy-integrating detectors (EID), integrate charges of all detected photons [16,17,18]. Willemink et al. give a detailed overview of the technical characteristics of PCDs [16].

Just as it is possible with CECT images acquired with DECT scanners, iodine and other contrast media can be selectively identified and virtually removed from CECT images acquired with PCCT scanners, resulting in VNC images. Due to an improved spectral separation, it is conceivable that VNC images obtained from a PCCT scanner could be more realistic compared to VNC images obtained from a DECT scanner [16,18].

Previous studies that describe the diagnostic value of VNC images based on DECT scanners [7,8,9,11]. Therefore, the purpose of the present study is to evaluate the diagnostic value and reliability of VNC images acquired with a PCCT scanner that is approved for clinical use. Subject of this study is a comparison of quantitative image properties of VNC and true non-contrast (TNC) images.

## 2. Material and Methods

### 2.1. Patient Population

Institutional review board approval was obtained. Informed consent was waived due to the retrospective study design. All scans were performed for diagnostic use with clinical standard protocols. Patient data were anonymized.

In total, 72 consecutive patients, who underwent a CT scan between September and October 2021, were retrospectively enrolled in this study. The demographic distribution of the patients was as follows: Total *n* = 72, mean age 67.8 years (range 39–91 years); female *n* = 36, mean age 66.3 years (range 39–84 years); male *n* = 36, mean age 69.3 years (range 42–91 years). Patients were not preselected regarding weight, age, sex or other characteristics.

### 2.2. CT Protocols and Image Acquisition

The patients underwent either a biphasic CT scan (non-enhanced scan as well as a contrast-enhanced scan in the portalvenous phase, *n* = 43) or a triphasic CT scan (additionally including a contrast enhanced scan in the arterial phase, *n* = 29). All CT examinations were performed with the PCCT Naeotom Alpha (software version Syngo CT VA40, Siemens Healthineers, Erlangen, Germany).

The examinations were performed for different clinical indications, including the assessment of malignant tumors, acute bleeding and others. Thus, scan parameters, such as contrast dosage and scan length, varied for different CT protocols and were not standardized. Iodinated contrast medium (Accupaque 300, GE Healthcare, Chicago, IL, USA) was administered in all examinations.

All examinations were performed in supine position. Tube voltage was 120 kVp and detector configuration was 144 × 0.4 mm^2^ with automatic tube current modulation. Scans were performed at a BQ-level of 170, pitch range of 0.8–2 and gantry rotation time of 0.25–0.5 s depending on the clinical protocol.

**Figure 1:**
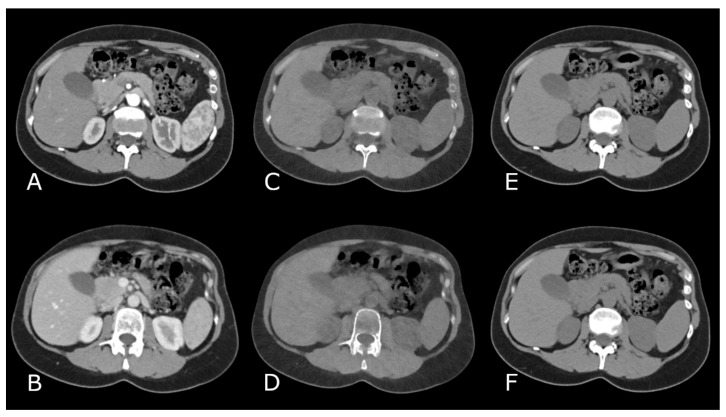
Images of a CT scan in arterial contrast phase (**A**) and in venous contrast phase (**B**) with corresponding virtual non-contrast images (**C**,**D**) and true non-contrast images from the same patient (**E**,**F**).

The datasets were analyzed retrospectively using a manufacturer specific spectral workstation (Syngo.Via, VB60 version, Siemens Healthineers, Erlangen, Germany). VNC images were reconstructed from the portalvenous phase (VNCv) as well as from the arterial phase (VNCa). All images were reconstructed in axial view with the same slice thickness (2 mm), image matrix (512) and iterative reconstruction level (Q4). TNC and VNC images were auto-registered by the prost-processing software (CT images are shown in Figure 1).

### 2.3. Quantitative Image Analysis

Regions of interest (ROI) were drawn in the following areas on VNCv, VNCa and TNC images: liver (5 cm^2^), spleen (2 cm^2^), kidney (0.3 cm^2^), aorta (0.5 cm^2^), (paravertebral) muscle (1 cm^2^), abdominal, subcutaneous fat (2 cm^2^) and spongious bone (vertebral body, 1 cm^2^). The size of the ROIs was initially adjusted to fit each tissue (as large as possible) and then kept constant in size in all datasets. The ROIs were placed in each area on the TNC images and then copied to the VNC images. If necessary, minimal manual adjustments were made to compensate for differences in breathing.

### 2.4. Statistical Analysis

Established software packages were used for the statistical analysis (SPSS Statistics 28, IBM, Armonk, NY, USA; Excel 2016, Microsoft, Redmond, WA, USA; R Core Team (2021). R: A language and environment for statistical computing. R Foundation for Statistical Computing, Vienna, Austria. URL https://www.R-project.org/ (accessed on 26 October 2021); RStudio Version 1.4.1106). If not stated otherwise, all data are presented as mean ± standard deviation of the mean (SD). Normal distribution was assumed based on sample size and histogram analysis. Significance of differences between VNC and TNC measurements was tested by two-sided paired t-test and Bland Altman plots for visualization. Pearson correlation coefficient was used to assess correlation between VNC and TNC measurements. *p*-values ≤ 0.05 were considered statistically significant.

## 3. Results

### 3.1. Comparison of TNC and VNC Images

**Figure 2:**
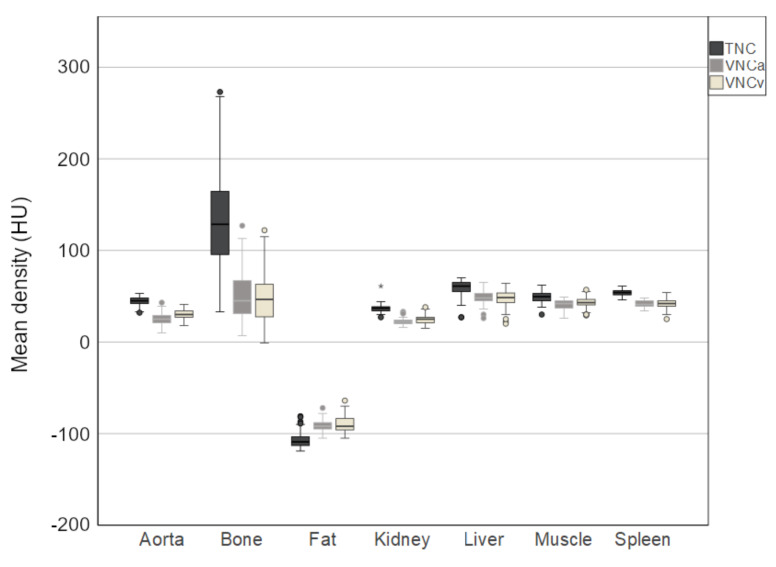
Boxplot showing the mean density in the different tissues measured on true non-contrast (TNC), virtual non-contrast images of a CT scan in arterial contrast phase (VNCa) and virtual non-contrast images of a CT scan in venous contrast phase (VNCv). Differences between measurements on TNC and VNCv and between TNC and VNCa images were found to be statistically significant for all tissues. The measured attenuation values were lower on VNCv and VNCa images compared to TNC images for aorta, bone, kidney, liver, muscle and spleen, while for fat attenuation values were less negative on VNCv and VNCa images compared to TNC. Differences between attenuation values on VNCv and VNCa images were only significant for liver tissue.

Mean attenuation HU (Hounsfield Units) ± SD of each tissue on TNC and VNC images is shown in Table 1. Mean offsets are presented in Table 2. There are significant differences between the attenuation on TNC and VNC images with attenuation being significantly lower in VNCv and VNCa images compared to TNC images for liver, spleen, kidney, aorta, muscle and bone. For Fat VNCv and VNCa images show less negative attenuation compared to TNC images (see also Figure 2).

The mean offset of attenuation values in spongious bone between TNC and VNC images was 86.13 HU ± 28.44 (VNCv) and 89.38 HU ± 32.69 (VNCa), respectively. There was only a small offset between VNCv and VNCa images (0.24 HU ± 3.93). Because of the clear difference between TNC and VNC images, these values were excluded from all following calculations.

#### 3.1.1. TNC versus VNCv

**Figure 3:**
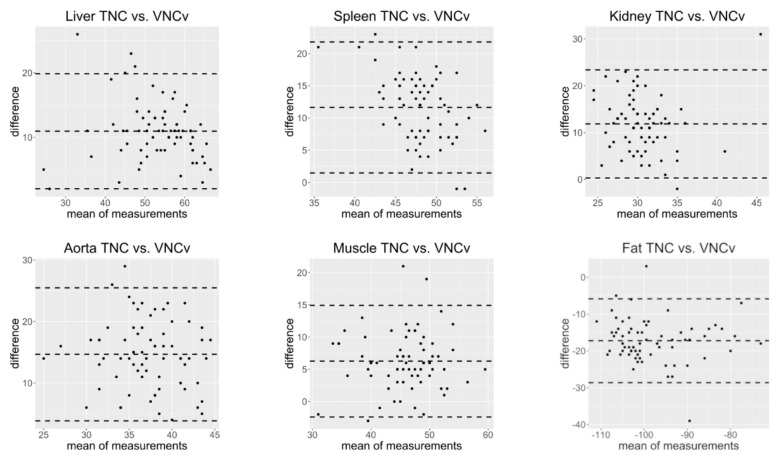
Bland Altman plots showing the relationship between the difference of measurements on true non-contrast (TNC) and virtual non-contrast images from a CT scan in venous contrast phase (VNCv) and the corresponding mean of measurements for the different tissues. All measurements spread around the mean of the differences and most measurements lie between ± 1.96 standard deviations of the difference. The difference is not substantially affected by the mean of measurements. For liver, spleen, kidney, aorta and muscle almost all measurements show a difference of >0 while for fat almost all measurements show a difference of <0.

The greatest offsets between absolute attenuation values of TNC and VNCv images were found in the aorta (14.67 HU ± 5.52, *p* < 0.001) and in fat (−17.24 HU ± 5.81, *p* < 0.001). The smallest offset was found in the muscle (6.26 HU ± 4.42, *p* < 0.001) and in the liver (10.96 HU ± 4.55, *p* < 0.001). The differences in attenuation values of TNC and VNCv images were statistically significant (*p* < 0.001) for all analyzed pairs (see Table 1). Bland Altman plots (Figure 3) show that almost all individual measurements show this tendency. The offset between attenuation values on VNCv and TNC is not substantially affected by the mean of both measurements (Figure 3).

Comparing TNC and VNCv images, 60% of all ROIs showed a difference of more than 10 HU and 28% showed a difference of more than 15 HU. The highest number of differences of more than 15 HU was found in fat (*n* = 46/72), the lowest number was found in muscle (*n* = 2/72).

**Figure 4:**
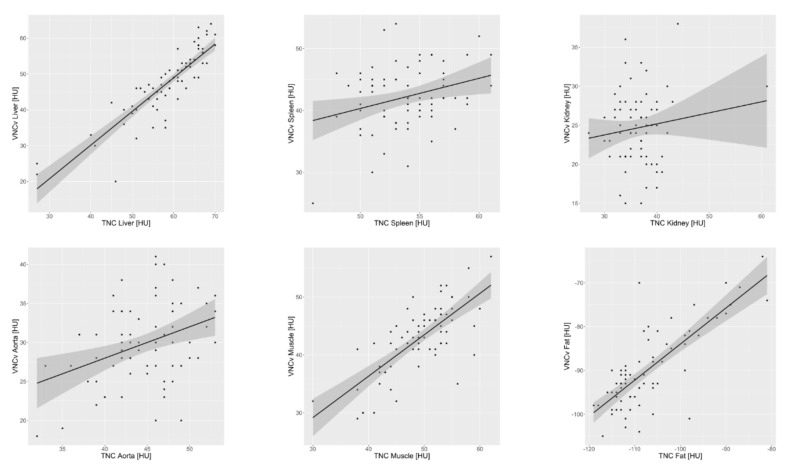
Scatterplots of tissue density on true non-contrast (TNC) and virtual non-contrast images from a CT scan in venous contrast phase (VNCv) for the different regions with linear regression lines and 95% confidence interval. A strong and significant correlation was found for liver, muscle, and fat (liver r = 0.876, *p* < 0.001/muscle r = 0.729, *p* < 0.001/fat r = 0.783, *p* < 0.001), while the correlation was only moderate but still significant for the aorta and spleen (aorta r = 0.368, *p* = 0.001/spleen r = 0.297, *p* = 0.011) and correlation was weak and not significant for the kidney (kidney r = 0.137, *p* = 0.25).

The attenuation values of liver, muscle and fat showed a strong and statistically significant correlation (TNC and VNCv: liver r = 0.876, *p* < 0.001/muscle r = 0.729, *p* < 0.001/fat r = 0.783, *p* < 0.001). At the same time, the attenuation values of spleen, kidney and aorta did not correlate strongly (TNC and VNCv: spleen r = 0.297, *p* = 0.011/kidney r = 0.137, *p* = 0.25/aorta r = 0.368, *p* = 0.001), even though the correlation was still significant for the spleen and the aorta (Figure 4).

#### 3.1.2. TNC versus VNCa

As on the VNCv images, the greatest offsets between TNC and VNCa images were found in the aorta (18.55 HU ± 8.10, *p* < 0.001) and in fat (−16.00 HU ± 4.43, *p* < 0.001). Likewise, the smallest offsets were found in the muscle (8.24 HU ± 4.25, *p* < 0.001) and in the liver (8.90 HU ± 3.11, *p* < 0.001). The differences in attenuation on TNC and VNCa images were statistically significant (*p* < 0.001) for all analyzed pairs as well (see Table 1).

In total, 64% of all ROIs on VNCa images showed a difference of more than 10 HU and 32% showed a difference of more than 15 HU compared to the TNC images. Different from the VNCv images, the highest number of differences of more than 15 HU on VNCa images was found in the aorta (*n* = 21/29), the lowest number was found in the liver (*n* = 0/29).

The attenuation values of liver, muscle and fat showed a strong and statistically significant correlation (TNC and VNCa: liver r = 0.938, *p* < 0.001/muscle r = 0.792, *p* < 0.001/fat r = 0.867, *p* < 0.001). At the same time, the attenuation values of spleen, kidney and aorta did not correlate strongly (TNC and VNCa: spleen r = 0.227, *p* = 0.237/kidney r = 0.394, *p* = 0.034/aorta r = 0.341, *p* = 0.07), but the correlation was still significant for the aorta.

#### 3.1.3. VNCv versus VNCa

**Figure 5:**
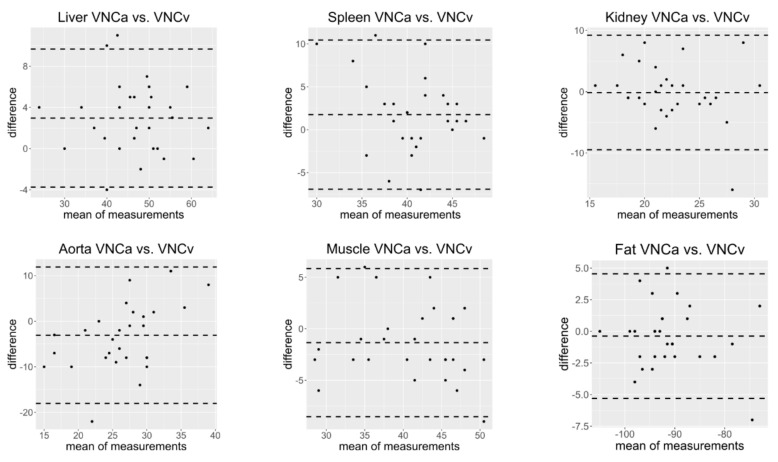
Bland Altman plots showing the relationship between the difference of measurements on virtual non-contrast images from a CT scan in arterial contrast phase (VNCa) and virtual non-contrast images from a CT scan in venous contrast phase (VNCv) and the corresponding mean of measurements for the different tissues. All measurements spread around the mean of the differences which is close to zero and most measurements lie between ± 1.96 standard deviations of the difference. The difference is not substantially affected by the mean of measurements.

The mean differences between the attenuation on VNCv and VNCa images were less than 4 HU in all tissues (see Table 2). In the hepatic tissue the difference between VNCv and VNCa images was significant (mean difference −2.97 HU ± 3.42, *p* < 0.001), whereas for all other tissues the differences were not statistically significant (*p* > 0.001, see Table 1). Bland Altman plots show that all measurements spread around the mean difference close to zero with no substantial influence of the mean of measurements. Almost all measurements lie between ± 1.96 SD (Figure 5).

A difference of more than 10 HU between corresponding VNCv and VNCa images was found in the liver (*n* = 1/29), the spleen (*n* = 1/29), the kidney (*n* = 1/29) and the aorta (*n* = 4/29); a difference of more than 15 HU only was found in the kidney (*n* = 1/29) and the aorta (*n* = 1/29).

**Figure 6:**
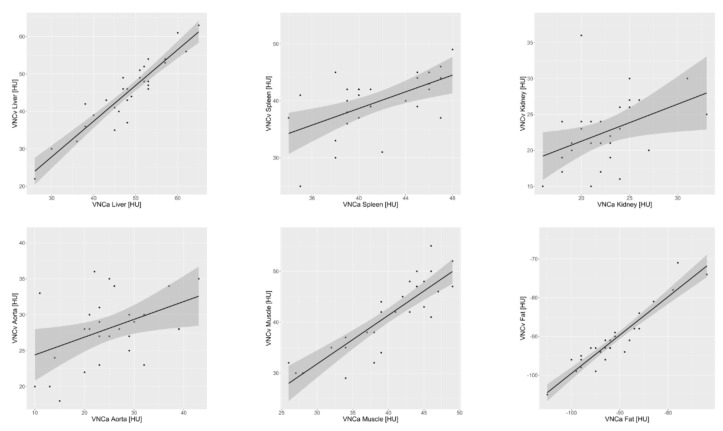
Scatterplots of tissue density on virtual non-contrast images from a CT scan in arterial contrast phase (VNCa) and virtual non-contrast images from a CT scan in venous contrast phase (VNCv) for the different regions with linear regression lines and 95% confidence interval. There was a significant correlation between attenuation values on VNCv and VNCa images for all tissues with the correlation being strong for liver, muscle, and fat, while the correlation was moderate for spleen, kidney, and aorta (VNCv and VNCa: liver r = 0.928, *p* < 0.001/muscle r = 0.862, *p* < 0.001/fat r = 0.941, *p* < 0.001/spleen r = 0.562, *p* = 0.001/kidney r = 0.413, *p* = 0.026/aorta r = 0.423, *p* = 0.022).

There was a significant correlation between attenuation values on VNCv and VNCa images for all tissues with the correlation being strong for liver, muscle, and fat, while the correlation was moderate for spleen, kidney, and aorta (VNCv and VNCa: liver r = 0.928, *p* < 0.001/muscle r = 0.862, *p* < 0.001/fat r = 0.941, *p* < 0.001/spleen r = 0.562, *p* = 0.001/kidney r = 0.413, *p* = 0.026/aorta r = 0.423, *p* = 0.022; Figure 6).

## 4. Discussion

The purpose of the present study was to evaluate the diagnostic reliability of VNC images acquired with the first PCCT scanner approved for clinical use, by comparing VNC images reconstructed from contrast enhanced CT scans and TNC images. A distinction was made between VNC images that were reconstructed from an arterial and a portalvenous contrast phase. Attenuation values were measured in liver, spleen, kidney, aorta, muscle, fat and spongious bone.

The acquisition of spectral datasets—the basis for iodine quantification and thus the reconstruction of VNC images from contrast enhanced CT scans—became possible with the introduction of the dual energy technology. Nowadays, this technology is well accepted and established in clinical routine. VNC images obtained with spectral CTs have been proven diagnostically reliable in various clinical studies [7,8,9,10]. Thus, it is conceivable that VNC images could potentially replace true non-enhanced phases [7], leading to a significant reduction in radiation dose.

The underlying algorithm for the subtraction of iodine in the present study provided a high reproducibility of attenuation values with only small differences between VNCv and VNCa images, which is in line with previous studies [7].

Studies using the dual energy technology serve as a comparison to the present study. However, it should be noted that the VNC images described in this study are based on datasets that were acquired using a different technology (PCD). Sauter et al. report about the reliability of VNC images obtained with an FDA and CE certified dual-layer CT scanner. They considered differences between TNC and VNC images of 10 HU or less as negligible, differences between 10 HU and 15 HU as acceptable. In their study, they describe a difference of 10 HU or less in over 80% and a difference of 15 HU or less in 92% of all measurements [7]. Toepker et al. tested the reliability of a dual energy CT and describe a difference of 10 HU or less in over 75.3% and a difference of 15 HU or less in 91.5% of all measurements [8]. Ananthakrishnan et al. describe similar results (difference less than 10 HU in 75.2% and less than 15 HU in 92.6% of all measurements) [9].

The results of the present study show differences of 10 HU or less between TNC and VNCv in 40% (VNCa 36%) and differences of 15 HU or less in 72% (VNCa 68%) of all measurements.

Despite these clear differences, the present study also shows parallels to previous studies. Even though the differences of absolute values in the present study are considerably higher, other studies also describe that the detected differences between the TNC and VNC images are statistically significant [7,8,9].

In addition, Sauter et al. and Toepker et al. both describe that they measured the highest differences between TNC and VNC images in the aorta and in fat, which is in line with the results of the present study [7,8]. It is argued that the difference between TNC and VNC images of the aorta may be related to the high concentration of iodine, particularly in the arterial phase [7]. The reason for the high difference of HU in fat remains unclear [7]. It should be assumed that due to the very low iodine uptake there should not be any significant difference between TNC and VNC images. Nevertheless, no explanation for this finding has been found yet [7].

The attenuation values of spongious bone on VNC images differed markedly from TNC. This problem is known from spectral CTs and has been described in previous studies [7,8,19]. The reconstruction of VNC images is based on material decomposition. Bone and iodine have a similar absorption profile. Therefore, bone is partially recognized as iodine and subtracted during the reconstruction process resulting in lower attenuation values of bone on VNC images. This finding was consistent on VNCv and VNCa images. Nevertheless, there was a strong correlation between TNC, VNCv and VNCa images suggesting a linear relationship. However, VNC images are not reliable for the assessment of spongious bone structure at this current state as the amount of mistakenly subtracted bone is unpredictable. Further studies are necessary to improve the algorithms and to make VNC images more reliable for the evaluations of bone structures.

There are certain limitations of the present study that have to be taken into consideration. Firstly, subjective image criteria are not considered, as the subject of this study is a comparison of objective image criteria. A qualitative evaluation of TNC and VNC images should be included in future studies. Secondly, the focus of this study is on quantitative image properties of TNC and VNC images for different anatomic tissues without regard to pathology. However, the diagnostic reliability of the VNC images should also be evaluated for different pathologies and clinical applications, e.g., the assessment of adrenal gland lesions or the contrast enhancement in liver or kidney tumors.

Although the results overlap in many ways with the experiences of previous studies based on dual energy technology, the mean differences between TNC and VNC images are considerably higher in the present study and the percentage of differences of 10/15 HU or less is considerably lower in the present study [7,8,9].

In conclusion, these findings clearly limit the diagnostic value of density measurements on VNC images at the current state. Nevertheless, the correlation between attenuation values on TNC and VNC images and the minor differences in attenuation values on VNCv and VNCa images suggest that the underlying algorithm for the subtraction of iodine works in principle, but with a systematic offset and requires further adjustments that the manufacturer will certainly provide as part of the upcoming updates. Until then, special caution should be exercised when using VNC images in routine clinical practice.

## Figures and Tables

**Table 1:** Mean attenuation HU ± SD of each tissue in TNC, VNCv and VNCa images.

	TNC	VNCv	TNC vs. VNCv	VNCa	TNC vs. VNCa	VNCv vs. VNCa
	Mean HU ± SD (*n* = 72)	Mean HU ± SD (*n* = 72)	Two-Sided *p*	Mean HU ± SD (*n* = 29)	Two-Sided *p*	Two- Sided *p*
Liver	58.58 ± 8.74	47.63 ± 9.38	<0.001	48.03 ± 8.88	<0.001	<0.001
Spleen	53.83 ± 3.14	42.19 ± 5.16	<0.001	41.55 ± 4.01	<0.001	0.042
Kidney	36.57 ± 4.41	24.69 ± 4.55	<0.001	22.34 ± 3.85	<0.001	0.877
Aorta	44.44 ± 4.69	29.78 ± 5.11	<0.001	25.07 ± 8.30	<0.001	0.039
Muscle	49.10 ± 6.05	42.83 ± 5.94	<0.001	40.21 ± 6.52	<0.001	0.058
Fat	−106.87 ± 8.56	−89.64 ± 9.02	<0.001	−91.31 ± 7.05	<0.001	0.423
Bone	133.44 ± 51.38	47.32 ± 25.70	<0.001	50.97 ± 30.51	<0.001	0.743

**Table 2:** Absolute differences of attenuation HU ± SD between TNC, VNCv and VNCa images and number of measurements with differences > 10/15 HU.

	TNC − VNCv			TNC − VNCa			VNCv − VNCa		
	Mean Offset HU ± SD	Diff. >15 HU	Diff. >10 HU	Mean Offset HU ± SD	Diff. >15 HU	Diff. >10 HU	Mean Offset HU ± SD	Diff. >15 HU	Diff. >10 HU
Liver	10.96 ± 4.55	10/72 (14%)	40/72 (56%)	8.90 ± 3.11	0/29 (0%)	7/29 (24%)	−2.97 ± 3.42	0/29 (0%)	1/29 (3%)
Spleen	11.64 ± 5.19	16/72 (22%)	42/72 (58%)	11.70 ± 4.52	5/29 (17%)	22/29 (76%)	−1.76 ± 4.44	0/29 (0%)	1/29 (3%)
Kidney	11.88 ± 5.88	15/72 (21%)	46/72 (64%)	14.97 ± 5.39	13/29 (45%)	24/29 (83%)	0.14 ± 4.76	1/29 (3%)	1/29 (3%)
Aorta	14.67 ± 5.52	31/72 (43%)	55/72 (76%)	18.55 ± 8.10	21/29 (72%)	26/29 (90%)	3.07 ± 7.64	1/29 (3%)	4/29 (14%)
Muscle	6.26 ± 4.42	2/72 (3%)	12/72 (17%)	8.24 ± 4.25	1/29 (3%)	9/29 (31%)	1.35 ± 3.67	0/29 (0%)	0/29 (0%)
Fat	−17.24 ± 5.81	46/72 (64%)	66/72 (92%)	−16.00 ± 4.43	16/29 (55%)	24/29 (83%)	0.38 ± 2.51	0/29 (0%)	0/29 (0%)
Total		120/432 (28%)	261/432 (60%)		56/174 (32%)	112/174 (64%)		2/174 (1%)	7/174 (4%)

## Data Availability

The data are available from the corresponding author on reasonable request.
